# A LAMP Assay for the Detection of *Thecodiplosis japonensis*, an Alien Gall Midge Species Pest of Pine Trees

**DOI:** 10.3390/insects13060540

**Published:** 2022-06-11

**Authors:** Jipeng Jiao, Lili Ren, Rumin Chen, Jing Tao, Youqing Luo

**Affiliations:** 1 Key Laboratory for Forest Pest Control, College of Forestry, Beijing Forestry University, Beijing 100083, China; jiao_jipeng666@bjfu.edu.cn (J.J.); lily_ren@bjfu.edu.cn (L.R.); 2 Sino-French Joint Laboratory for Invasive Forest Pests in Eurasia, Beijing Forestry University-French National Research Institute for Agriculture, Food and Environment (INRAE), Beijing 100083, China; 3 Huangdao District Natural Resources Bureau, Qingdao 266520, China; crm630713@163.com

**Keywords:** pine needle gall midge, *Thecodiplosis japonensis*, *COI*, loop-mediated isothermal amplification (LAMP), field diagnostics

## Abstract

**Simple Summary:**

*Thecodiplosis japonensis* is considered the most harmful pest to pines in South Korea. *T. japonensis* is a native species of Japan. Recently, *T. japonensis* was discovered in China and has caused serious damage to local pine trees. Due to the small size and little morphological difference with its related species, it is difficult to accurately identify *T. japonensis* by morphological methods. Accurate and efficient molecular identification methods are urgently needed to detect this invasive gall midge pest, yet there was no molecular identification method for *T. japonensis*. In this study, we developed a LAMP assay to detect *T. japonensis* based on the *COI* gene sequence. The LAMP assay could detect as little as 300 fg of gDNA. Using colorimetric amplification and a crude gDNA extraction method, the total procedure could be processed in 75 min. The method established in the study can be easily used in both laboratory and field conditions, enabling rapid molecular identification of *T. japonensis*.

**Abstract:**

Pine needle gall midge (*T. japonensis*), native to Japan, has become a serious invasive pest in South Korea and, more recently in 2006, in China. It was first discovered in Qingdao, Shandong Province, and has caused serious damage to local *Pinus thunbergii*. The insect’s small size makes morphological-based identification difficult; therefore, molecular detection techniques are urgently needed for monitoring and preventing its further spread. At present, there is no simple and accurate field molecular identification tool. To solve this problem, a LAMP-based molecular diagnosis technology of *T. japonensis* was developed. Four LAMP primers were designed to specifically amplify *T. japonensis* DNA. Positive LAMP reactions usually produce amplification in one hour. The optimal incubation conditions for LAMP detection were determined with 4 LAMP primers for 60 min at 61 °C. The LAMP detection range of gDNA concentrations is wide, with a minimum detectable gDNA concentration of 300 fg. A non-destructive DNA-releasing procedure, HotSHOT “HS6”, which could extract “crude DNA” for LAMP assay in 10 min, was used for larval and adult samples. Therefore, we established a LAMP-based rapid molecular identification method that can be applied in the monitoring and management of *T. japonensis*.

## 1. Introduction

Pine needle gall midge, *T. japonensis* (Uchida and Inouye) (Diptera: Cecidomyiidae), is a native pest of Japan. *T. japonensis* belongs to the Cecidomyiidae family, which contains 6651 known species and 832 genera [1]. The family is divided into six subfamilies. The largest subfamily, the Cecidomyiinae, best known as herbivores and plant gall makers, such as pine needle gall midge, includes some of the most destructive pests of grains, fruits and vegetables and also many fungivores and predators of plant-feeding arthropods [1,2,3]. In addition to *T. japonensis*, there are another five species in the genus *Thecodiplosis* recorded: *Thecodiplosis brachyntera* in Europe; *Thecodiplosis brachynteroides*, *Thecodiplosis pinirigidae*, *Thecodiplosis piniradiatae*, and *Thecodiplosis piniresinosae* in North America. These gall midges broke out locally from the 1930s to the 1990s [4,5,6]. However, there have been no reports of spread and harm caused by them in the past two decades.

First discovered in South Korea in 1924, *T. japonensis* has widely spread throughout the country South Korea [7]. Discovered in China in 2006, the tiny invasive pest has seriously endangered *P. thunbergii* Parlatore, *Pinus densiflora* Sieb. et Zucc. and *Pinus tabuliformis* Carriere [8]. It is considered the most harmful pest to pines in South Korea, causing great damage to the South Korean ecological environment and economic development [9,10]. In Japan, *T. japonensis* could attack *Pinus massonian* Lambert [11]. *T. japonensis* is mainly spread by humans over long distances with the transfer of host plants or soil-carrying plants, and natural dispersion mainly depends on the flight of adults [12]. *T. japonensis* is an univoltine species in Shandong Province. Larvae hibernate in the soil where they pupate in late May. Adults emerge from the soil from the end of late May until July. After mating, females search for a suitable host plant and lay eggs on needle pairs of current-year shoots in the vicinity of where they emerged from the soil. Larvae develop inside galls at the base of needle pairs. In the autumn, they leave the galls and drop to the soil where they pupate and remain until the following year [8,13]. Galls are formed when larvae feed on the needles, stopping needle growth and causing them to become shorter than healthy needles, which then gradually wither and die [14,15].

In China, there is a pest of the same genus that infests *P. massoniana,* mainly in Fujian Province. Since its larval and adult morphological characteristics are almost the same as *T. japonensis*, it has long been misidentified as *T. japonensis* [16] until Jiao separated the two by larval morphology and mitochondrial gene differences [13]. These two gall midges cause similar damage to their host plants [8,16]. Specialized entomological knowledge is needed to accurately distinguish them [13,16]. Therefore, to improve monitoring, early detection and pest management of *T. japonensis*, an effective identification method is urgently needed. However, there is no molecular detection method for *T. japonensis* so far. In addition to the morphological method for *T. japonensis* identification, there is an urgent need for rapid detection methods that can be used for field detection.

At present, the molecular methods for identifying similar pests include DNA barcoding based on *COI* gene, real-time qPCR and so on. Loop-mediated isothermal amplification (LAMP) is a one-step nucleic acid amplification technology based on auto-cycling strand-displacement DNA synthesis and could be undertaken in the field without high equipment and personnel [17,18,19,20,21,22]. In general, the LAMP method only requires a hot block to amplify DNA, and the result is detectable by the naked eye. This method has been widely used in the detection of many kinds of pests, such as *Myzus persicae*, *Spodoptera frugiperda*, etc. [23,24,25,26,27,28].

In our study, a sensitive, specific LAMP assay for the rapid identification of *T. japonensis* was developed. A rapid DNA extraction method that can be used in the field is applied to save time. Colorimetric LAMP assay requires only a portable heating block for clear visual detection with the naked eye. We also designed a pair of *T. japonensis* species-specific primers based on the *COI* gene sequences and compared the results of two diagnostic methods.

## 2. Materials and Methods

### 2.1. Samples Examined

During the adult emergence period, a net was used to catch the mating adults of *T. japonensis* and *Thecodiplosis* sp. on the grass under the infested pine tree. The infected pine trees were identified by searching the galls formed on shortened needles of the previous year. Individual larvae of *T. japonensis* and *Thecodiplosis* sp. were obtained in galls formed on current-year needles before they left the galls and dropped to the soil. Other non-target gall midge specimens collected are listed in Table 1. Specimens were confirmed by DNA barcoding of *COI* using a pair of universal primers (C1-J1709 and C1-N2353) [29] and a morphological method. Molecular phylogenetic analysis was carried out using the neighbor-joining method by MEGA 7. The *COI* sequences of other gall midges were downloaded from NCBI (see Supplementary File S1) and compared to the reference sequence of *Drosophila melanogaster* and *Aedes aegypti*.

Genomic DNA (gDNA) was extracted from adults and larvae using the TIANamp Micro DNA Kit (TIANGEN, Beijing, China), following the manufacturer’s instructions. The extracted DNA was quantified by a spectrophotometer (Thermo Fisher, Waltham, MA, USA) and stored at −20 °C. Another “crude” DNA extraction method that is non-destructive to specimens was tested in the LAMP assay. An individual gall midge larva or adult was processed according to a modified HotSHOT protocol [30]. Briefly, a single larval or adult was placed in 20 μL of premixed TE buffer, pH 8.0 (Invitrogen, Waltham, MA, USA) and 25 mmol L-1 NaOH (1:1), and denatured at 95 °C for 10 min, followed by > 1 min incubation on ice. DNA was stored at −20 °C.

### 2.2. LAMP Primer Design

Based on the analysis of mitochondrial genome sequences (unpublished data) from *T. japonensis*, *Thecodiplosis* sp. and mitochondrial genome sequences in NCBI from three other gall midges (*Mayetiola destructor*, *Orseolia oryzae* and *Rhopalomyia pomum*), a partial *COI* sequence region was chosen as a target region to design LAMP primers. Nine *COI* sequences containing the target region were downloaded from GenBank and NCBI (see Supplementary File S2) from the closely related gall midges. For comparative analysis, *COI* sequences were aligned using MEGA 7. Six LAMP primers were manually designed targeting eight DNA regions and synthesized by a commercial company (SinoGenoMax, Beijing, China). Potential primer–dimer interactions of LAMP primers were analyzed.

### 2.3. LAMP Assay

A WarmStart^®^LAMP Kit (New England Biolabs, Ipswich, MA, USA) was used for the LAMP assay. The general LAMP protocol followed the manufacturer’s guidelines using a 25 μL reaction mixture. Each reaction contains 4 primers (outer primer F3 and B3, and inner primer FIP and BIP) and 30 ng of gDNA. Two additional loop primers (Floop and Bloop) were tested to reduce the incubation time. Different reaction temperatures (range 59 to 63 °C) were tested to determine the optimum working conditions. LAMP assays were performed on a CFX96 thermocycler (BIO-RAD, Hercules, CA, USA), and the amplification curves could be visualized on the thermocycler screen. The results could also be checked under UV light with GelRed (BIOTIUM, Fremont, CA, USA), visible light or upon gel electrophoresis.

### 2.4. Analytical Detection Limit of the LAMP Assay

DNA extract from *T. japonensis* was ten-fold serial diluted using ultrapure water (Invitrogen, Waltham, MA, USA). Starting DNA concentration was quantified by a spectrophotometer (Thermo Fisher, Waltham, MA, USA). The DNA concentration was diluted from 30 ng/µL to 3 fg/µL serially. Detection limit of the LAMP assay was tested following the same assay conditions as mentioned above using the serially diluted DNA. The results could be checked under UV light with GelRed (BIOTIUM, Fremont, CA, USA), visible light or upon gel electrophoresis.

### 2.5. Species-Specific PCR Assay

A pair of *T. japonensis*-specific primers, named *COI*-F and *COI*-R, were designed by Primer 5.0 according to the difference in *COI* gene sequence between *T. japonensis* and related gall midges. The Primer-BLAST found in the NCBI database showed that the primer set only matched the *COI* gene fragment of *T. japonensis*. The primers were synthesized by a commercial company (SinoGenoMax, Beijing, China). For SS-*COI* PCR, Prime Star Mix (Tsing Ke, cat#R045) was used together with two SS-*COI* primers (*COI*-F and *COI*-R) following the protocol: 94 °C for 2 min followed by 35 cycles of 94 °C for 30 s, 61°C for 30 s and 72 °C for 1 min. The serial dilution of gDNA extracts was the same as the LAMP assay. PCR fragments were separated by 1.0% agarose gel and checked under UV light with GelRed.

## 3. Results

### 3.1. Specimens Examined

**Figure 1:**
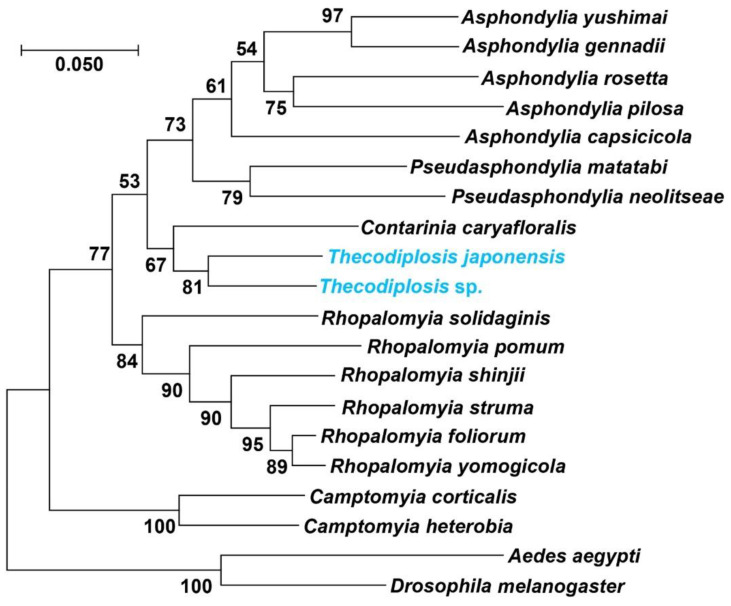
Phylogenetic tree reconstructed using NJ based on *COI* sequence.

All specimens examined were confirmed by DNA barcoding of *COI* and the morphological method in the current study (Table 1). The *Thecodiplosis* sp. was the most homologous species of *T. japonensis* in NCBI, which showed high similarity by MegaBLAST. The phylogenetic relationship between *COI* genomes of 18 species was examined (Figure 1), and the result of the phylogenetic relationship was similar to the megaBLAST result.

### 3.2. Development of the LAMP Assay

**Figure 2:**
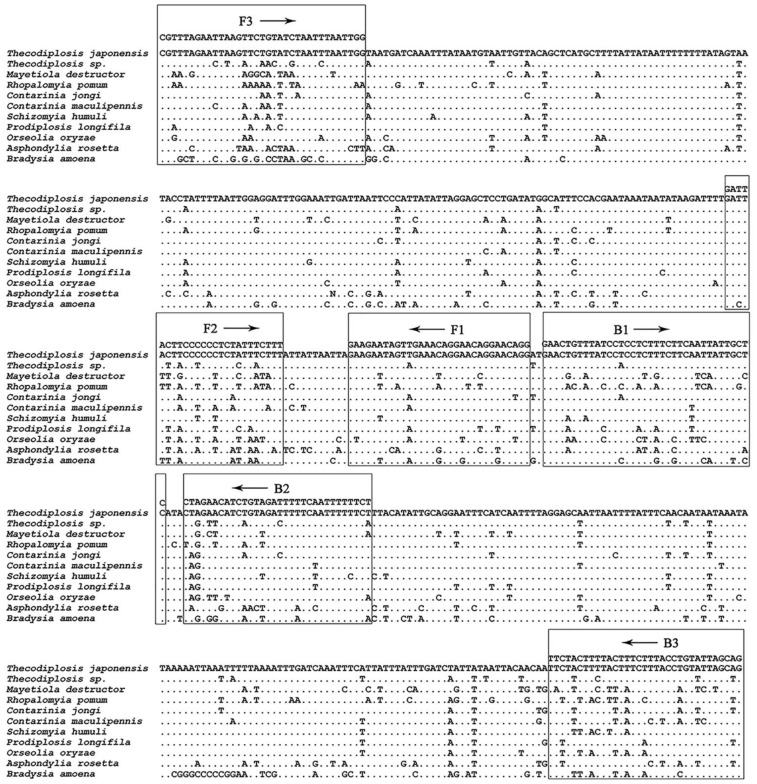
Alignment of *COI* gene sequences of *T. japonensis* with related gall midges. Primer regions are marked with boxes. FIP is composed of the reverse complement of F1 and F2; BIP is composed of B1 and the reverse complement of B2.

Four LAMP primers (Table 2), including two outer primers of Tjap_F3 and Tjap_B3, and two inner primers of Tjap_FIP and Tjap_BIP (Figure 2), were designed manually by aligning *COI* gene sequences of *T. japonensis* and related gall midges. The primer ratio of outer primers and inner primers was tested, and the optimal primer ratio was determined to be 1:8, with final concentrations of 0.2 μM and 1.6 μM, respectively (Table 3). LAMP assay was tested in a CFX96 thermocycler at various incubation temperatures of 59~63 °C. The optimal reaction condition was incubated at 61 °C for 60 min. Two loop primers of Tjap_FL/Tjap_BL were tested in a CFX96 thermocycler (BIO-RAD, Hercules, CA, USA) for acceleration of the reaction [31]. However, there was no significant increase in amplification efficiency by adding Tjap_FL and Tjap_BL.

### 3.3. Performance of the LAMP Assay

**Figure 3:**
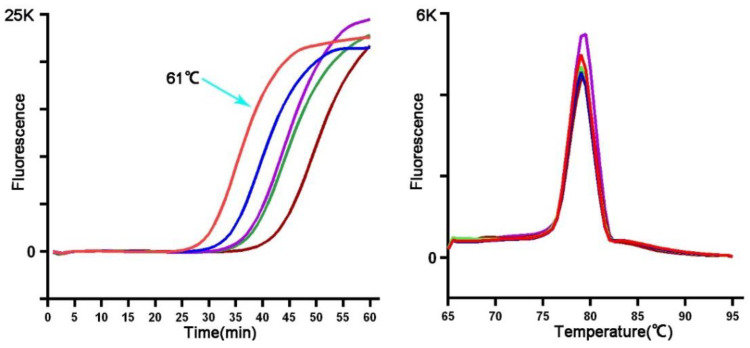
Different reaction temperatures (range: 59 to 63 °C) were tested to determine the optimal reaction temperature of LAMP. The optimal incubate condition was 61 °C for 60 min with an anneal derivative temperature of 79 °C.

**Figure 4:**
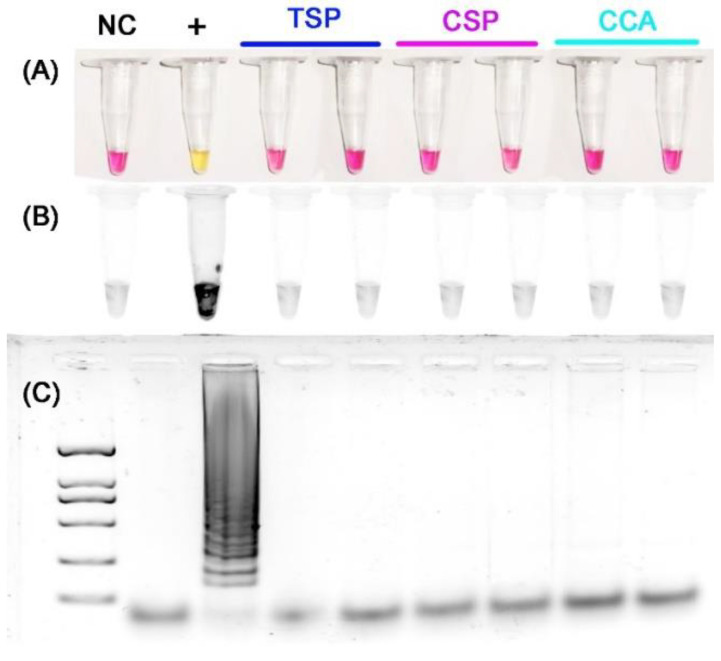
Specificity of the LAMP assay with four LAMP primers. (**A**) Checking results under visible light, (**B**) checking results under ultraviolet light with GelRed and (**C**) checking results by gel electrophoresis. NC: negative control, +: *T. japonensis*, TSP: *Thecodiplosis* sp., CSP: *Contarinia* sp., CCA: *C. caryafloralis*.

Usually, the LAMP assay amplifies the gDNA of *T. japonensis* within 60 min, and the anneal derivative temperature is approximately 79 °C (Figure 3). All non-target gall midge species, including the most closely related *Thecodiplosis* sp., did not amplify. The positive reaction can be checked under visible light with the color change from red to yellow, confirmed by gel electrophoresis or under UV light with GelRed (Figure 4).

**Figure 5:**
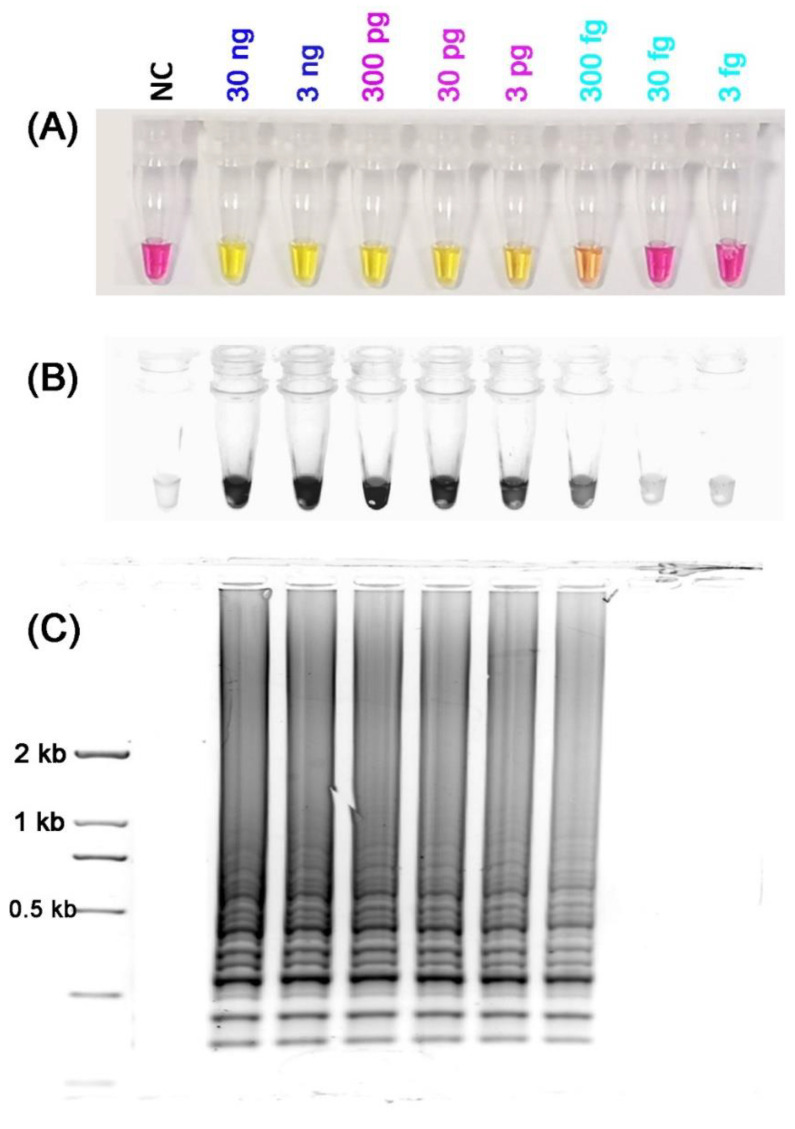
Identification of the detection limit of gDNA in the LAMP assay from 30 ng to 3 fg. (**A**) Checking results under visible light, (**B**) checking results under ultraviolet light with GelRed and (**C**) checking results by gel electrophoresis. NC, non-template control.

The detection limits of the LAMP assay were tested using the “clean” DNA (from a standard DNA isolation protocol). Whether under visible light, using ultraviolet light or gel electrophoresis, the four LAMP primers could detect as little as 300 fg of gDNA (Figure 5).

**Figure 6:**
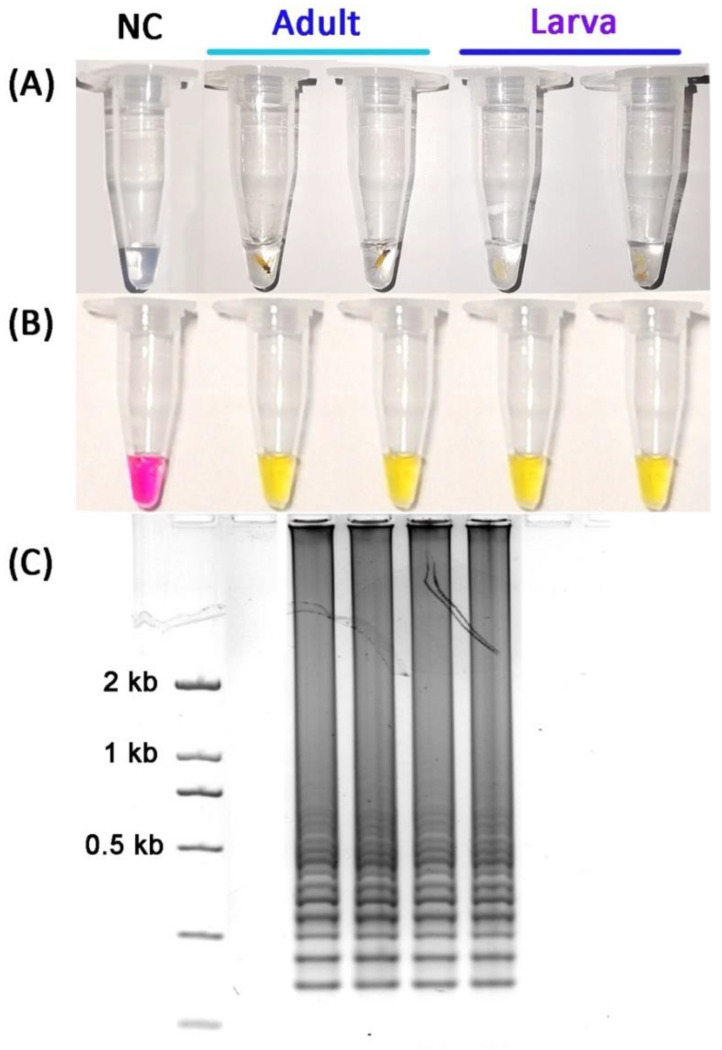
Identification of the “crude” gDNA extract using a non-destructive DNA extraction method with four LAMP primers. (**A**) Individual larva and adult, (**B**) under visible light, (**C**) under gel electrophoresis.

The non-destructive DNA extraction method from whole specimens was applied to obtain *T. japonensis* gDNA from single adult and larva, suitable for the LAMP assay. A colorimetric LAMP assay was conducted to test this “crude” gDNA extraction method in a heating block. The result was also checked by gel electrophoresis. Both adult and larval samples of *T. japonensis* generated positive results within 60 min (Figure 6), with 100% success rates when 10 samples per adult and larva were tested. This allows the preservation of complete morphological voucher specimens for further species identification by molecular or morphological identification method.

### 3.4. Performance of Species-Specific PCR Assay

**Figure 7:**
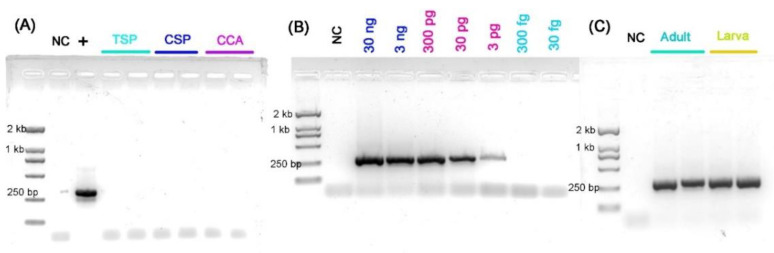
Species-specific PCR assay results with two SS-*COI* primers. (**A**) Species-specificity test of *T. japonensis* SS-*COI* primers. (**B**) Identification of the detection limit of gDNA in the species-specific PCR assay from 30 ng to 30 fg. (**C**) Species-specific PCR assay results with genomic DNA extracted from larvae and adults. NC: negative control, +: *T. japonensis*, TSP: *Thecodiplosis* sp., CSP: *Contarinia* sp., CCA: *C. caryafloralis*.

The specificity of the species-specific primer pair *COI*-F and *COI*-R was tested, resulting in a 299-bp fragment obtained from the gDNA of *T. japonensis*, with no amplification detected for the three other gall midges. Sensitivity tests were performed on larvae and adults. The detection limit of the species-specific PCR assay was 3 pg of *T. japonensis* gDNA (Figure 7).

## 4. Discussion

In recent years, *T. japonensis* have caused serious damage to *P. thunbergii* in Shandong, China. In South Korea, *T. japonensis* have been reported for nearly 100 years and have caused serious ecological and economic losses [9,10]. Inoculation tests showed that *T. japonensis* could infest a variety of pine trees, including *P. massoniana*, *P. resinosa*, *P. thunbergii*, *Pinus sylvestris*, *Pinus mugo*, *Pinus luchuensis*, *Pinus tabulaeformis*, *Pinus radiata*, *Pinus coulteri*, *Pinus nigra*, *P. densiflora*, *Pinus taiwanensis* and *Pinus insularis* [15].

The potential geographical distribution of *T. japonensis* in China was predicted by a MaxEnt niche model and CLIMEX model (unpublished data). The results showed that most areas of southern and central China were highly suitable areas for *T. japonensis* establishment and outbreak, coinciding with a wide distribution of pine trees that inoculation tests showed could be infested by *T. japonensis*. Thus, the risk of *T. japonensis* spreading in China, and even globally, is high.

Early detection and management of invasive pests are crucial. It is difficult to accurately identify *T. japonensis* by traditional morphological methods due to the small size and little morphological difference with its related species. The small shape difference of sternal spatula (width no more than 50 microns) on the pronotum of mature larvae and the wing vein of adults are key identification plural of genus *Thecodiplosis*. Jiao used the geometric morphology method to identify *T. japonensis* by analyzing the shape of the sternal spatula of larvae [13]. This morphological identification method requires a series of operations such as making slide specimens, micrographs and picture analysis with professional software, which is extremely time-consuming and requires trained professionals. Accurate and efficient molecular identification methods are urgently needed to detect this invasive gall midge pest, yet there was no molecular identification method for *T. japonensis*.

LAMP has higher sensitivity and amplification efficiency than other molecular identification methods, and its results can be visually monitored either through a color change of fluorescent intercalated dye or turbidity. Recently, LAMP has been widely used in many fields, such as ecology, medical research and also to diagnose mutations associated with resistance in insects [23,32]. The accuracy, simplicity and high-throughput adaptability of LAMP analysis are advantageous [33,34,35]. In this study, a LAMP method for on-site identification of *T. japonensis* was developed. We designed a new set of specific LAMP primers based on the *COI* sequence. In the LAMP assay, the detection limit of four primers was 300 fg gDNA within 60 min. The optimized method is fast, stable and specific, and even the most closely related *Thecodiplosis* sp. did not produce positive amplification. Two loop primers (Tjap_FL and Tjap_BL) were used to reduce the incubation time. However, there was no significant increase in amplification efficiency by adding both Tjap_FL and Tjap_BL or adding them separately.

It is essential that the field detection of LAMP obtains a fast, reliable and simple DNA extraction method. Nowadays, some non-destructive DNA extraction methods are used to obtain insect DNA for molecular experiments [36]. The published HotSHOT protocol provides a simple and rapid method for obtaining genomic DNA for LAMP assay. Genomic DNA can be successfully obtained by simple incubation of specimens in NaOH and Tris mixed solution by HotSHOT protocol. The specimens extracted by this non-destructive method still retain the morphological characteristics, which can be used for morphological identification for further confirmation. Thus, in our study, even without special equipment such as a PCR machine or electrophoresis equipment, DNA can be detected from adult and larvae specimens within 75 min using only hot blocks. Besides LAMP, we also developed a PCR method based on a mitochondrial DNA *COI* marker to detect *T. japonensis*. According to the difference in *COI* gene sequence between *T. japonensis* and related gall midges, a pair of *T. japonensis*-specific primers were designed. The specificity of the primers was tested on related gall midges. The detection limit of this method was 3 pg gDNA of *T. japonensis*. Thus, the two molecular detection methods can be applied to the identification of *T. japonensis*. Each method provides a different advantage depending on the situation. The LAMP assay is appropriate in field situations or for urgent samples, while the SS-*COI* PCR method is more suitable for a large number of sample extracts, without a time limit, in a laboratory setting with experienced personnel.

In this study, it was the first time that LAMP was used for molecular detection of cecidomyiid insects. It is worth further investigation to determine whether other congeners such as *T. brachyntera* and *T. brachynteroides*, which have not been tested in this study due to the unavailability of DNA samples, could amplify their gDNA using the LAMP assay.

## 5. Conclusions

In this study, a LAMP assay was successfully developed to detect the invasive pest *T. japonensis* based on the *COI* gene sequence. Using colorimetric amplification and crude gDNA extraction method, the total procedure could be processed in 75 min. Specific primers, optimized LAMP reaction temperatures and easily timed steps make the method specific, stable and reproducible. Therefore, the LAMP assay could be used to detect *T. japonensis* in field-collected populations. The new method established in the study is a portable, rapid molecular identification tool for *T. japonensis*, which can be easily used in both laboratory and field conditions, enabling rapid molecular identification of *T. japonensis*.

## Figures and Tables

**Table 1:** Specimens used in LAMP assay and SS-COI PCR to identify *T. japonensis* from other gall midges.

Genus	Species	Stage and Amount	Collection Date	Location Information
*Thecodiplosis*	*T. japonensis*	Adult/Larvae (58/52)	June 2020	Shandong China (33°230′18″ N, 126°370′40″ E)
	*Thecodiplosis* sp.	Adult/Larvae (24/98)	March 2020	Fujian China (24°37′29″ N, 116°54′40″ E)
*Contarinia*	*Contarinia* sp.	Larvae (21)	April 2018	Xinjiang China (41°9′34″ N, 80°10′14″ E)
	*Contarini caryafloralis*	Larvae (85)	August 2018	Anhui China (30°20′23″ N, 118°44′23″ E)

**Table 2:** Primers for LAMP and SS-*COI* PCR.

Assay	Primer Name	Sequence (5′-3′)
LAMP	Tjap_F3	CGTTTAGAATTAAGTTCTGTATCTAATTTAATTGG
	Tjap_B3	CTGCTAATACAGGTAAAGAAAGTAAAAGTAGAA
	Tjap_FIP	CCTGTTCCTGTTCCTGTTTCAACTATTCTTCGATTACTTCCCCCCTCTATTTCTTT
	Tjap_BIP	GAACTGTTTATCCTCCTCTTTCTTCAATTATTGCTCAGAAAAAATTGAAAAATCTACAGATGTTCTAG
	Tjap_LF	CCTGTTCCTGTTTCAACTATTCTTCTAATTAATAAT
	Tjap_LB	GTTTATCCTCCTCTTTCTTCAATTATTGCTCATA
SS-*COI* PCR	COⅠ-F	CAGGTAAAGAAAGTAAAAGTAGAATTGTTGTAATT
	COⅠ-R	GATTTTGATTACTTCCCCCCTCTATTTC

**Table 3:** Optimized LAMP components for the detection of *T. japonensis*.

LAMP Components	Concentrations Used for Optimization	Final Concentrations Used in LAMP
WarmStart^®^LAMP Master Mix	2×	1×
F3	10 μM	0.2 μM
B3	10 μM	0.2 μM
FIP	10 μM	1.6 μM
BIP	10 μM	1.6 μM
DNA sample	Variable	Variable

## Supplementary Materials

The following supporting information can be downloaded at: https://www.mdpi.com/article/10.3390/insects13060540/s1, File S1: *COI* sequences used in molecular phylogenetic analysis; File S2: *COI* sequences used in LAMP primers design.
